# Detecting low blood concentrations in joints using T1 and T2 mapping at 1.5, 3, and 7 T: an *in vitro* study

**DOI:** 10.1186/s41747-021-00251-z

**Published:** 2021-12-02

**Authors:** Flora H. P. van Leeuwen, Beatrice Lena, Jaco J. M. Zwanenburg, Lize F. D. van Vulpen, Lambertus W. Bartels, Kathelijn Fischer, Frank J. Nap, Pim A. de Jong, Clemens Bos, Wouter Foppen

**Affiliations:** 1 Department of Radiology, Division of Imaging and Oncology, University Medical Center Utrecht, Utrecht University, Heidelberglaan 100, 3584 CX Utrecht, The Netherlands; 2 Image Sciences Institute, University Medical Center Utrecht, Utrecht University, Heidelberglaan 100, 3584 CX Utrecht, The Netherlands; 3 Van Creveldkliniek, University Medical Center Utrecht, Utrecht University, Heidelberglaan 100, 3584 CX Utrecht, The Netherlands; 4 Department of Radiology, Central Military Hospital, Ministry of Defence, Lundlaan 1, 3584 EZ Utrecht, The Netherlands; 5 Division of Imaging and Oncology, University Medical Center Utrecht, Utrecht University, Heidelberglaan 100, 3584 CX Utrecht, The Netherlands

**Keywords:** Haemarthrosis, Image interpretation (computer-assisted), Magnetic resonance imaging, Phantoms (imaging), Synovial fluid

## Abstract

**Background:**

Intra-articular blood causes irreversible joint damage, whilst clinical differentiation between haemorrhagic joint effusion and other effusions can be challenging. An accurate non-invasive method for the detection of joint bleeds is lacking. The aims of this phantom study were to investigate whether magnetic resonance imaging (MRI) T1 and T2 mapping allows for differentiation between simple and haemorrhagic joint effusion and to determine the lowest blood concentration that can be detected.

**Methods:**

Solutions of synovial fluid with blood concentrations ranging from 0 to 100% were scanned at 1.5, 3, and 7 T. T1 maps were generated with an inversion recovery technique and T2 maps from multi spin-echo sequences. In both cases, the scan acquisition times were below 5 min. Regions of interest were manually drawn by two observers in the obtained T1 and T2 maps for each sample. The lowest detectable blood concentration was determined for all field strengths.

**Results:**

At all field strengths, T1 and T2 relaxation times decreased with higher blood concentrations. The lowest detectable blood concentrations using T1 mapping were 10% at 1.5 T, 25% at 3 T, and 50% at 7 T. For T2 mapping, the detection limits were 50%, 5%, and 25%, respectively.

**Conclusions:**

T1 and T2 mapping can detect different blood concentrations in synovial fluid *in vitro* at clinical field strengths. Especially, T2 measurements at 3 T showed to be highly sensitive. Short acquisition times would make these methods suitable for clinical use and therefore might be promising tools for accurate discrimination between simple and haemorrhagic joint effusion *in vivo*.

**Supplementary Information:**

The online version contains supplementary material available at 10.1186/s41747-021-00251-z.

## Key points

- T1 and T2 mapping can differentiate simple and haemorrhagic effusion *in vitro*.
- T1 and T2 relaxation times decreased as the blood concentration increased.
- The lowest blood concentrations detected using T1 mapping was 10% at 1.5 T.
- The lowest blood concentrations detected using T2 mapping was 5% at 3 T.

## Background

Haemarthrosis may be caused by (repeated) trauma or by bleeding disorders like haemophilia and Von Willebrand disease. The intra-articular blood has harmful effects on different joint components [1, 2]. These harmful effects already occur after relatively short exposure to small amounts of intra-articular blood as shown *in vitro* [3].

Clinical symptoms of joint bleeding, such as pain, swelling, and reduced functionality, are not specific for joint bleeding as these are observed in different joint conditions like arthritis, osteoarthritis, and other arthropathies as well [4–6]. Identification of joint effusion by imaging studies for example is not specific for joint bleeds, because effusion is also observed in healthy joints [7], arthropathy flare-ups, and arthritis [4, 8–10]. Nevertheless, being able to distinguish between a joint bleed and another cause of joint effusion is clinically relevant, especially in patients with bleeding disorders like haemophilia, since the different diagnoses require different treatments [4, 11, 12].

The current reference standard for the analysis of joint effusion is a joint aspiration. However, it is an invasive method that could introduce the potential risk of infection or a haemorrhagic procedure which could result in false-positive outcomes. Therefore, joint aspiration might not be desirable in all cases, e.g*.* in patients with an increased bleeding risk [11, 13].

Ultrasound is increasingly used in addition to physical examination since it is a non-invasive method that can accurately assess joint effusion and changes in synovial tissue [11, 14–16], it is widely available, and provides real-time information [11, 14–16]. Moreover, previous studies have shown that ultrasonography is sensitive to differentiate between simple and complex joint effusion [17, 18]. However, limitations of ultrasound are the operator dependency and therefore a substantial inter-observer variation of findings [11, 19].

Magnetic resonance imaging (MRI) is a reliable tool for the assessment of inflammatory and degenerative joint changes, due to its excellent contrast in tissues and fluids [10, 14]. Large haemarthroses may show fluid-fluid levels or thrombi on MRI. However, the accuracy of standard MRI protocols for the detection of early or minor joint bleeding has not been established yet [17, 18]. Therefore, an accurate non-invasive reference standard to detect joint bleeding is lacking.

Whilst standard MRI protocols are unable to accurately detect limited amounts of intra-articular blood, dedicated MRI sequences might be able to detect and quantify small amounts of blood within the synovial fluid. Since synovial fluid and iron-containing blood have different relaxation properties, quantification of longitudinal (T1) and transverse (T2) relaxation times with T1 and T2 mapping should in principle be able to assess the presence of blood in the synovial fluid.

In this phantom study, we investigated the potential of quantitative T1 and T2 relaxometry MRI protocols as non-invasive tools to differentiate between simple and haemorrhagic joint effusion at 1.5, 3, and 7 T. Specifically, we aimed to establish the minimal blood concentration in the synovial fluid which can be detected using these quantification methods.

## Methods

### Sample preparation

All patients or their legal guardians approved the use of their remnant samples for method development, validation, and research purposes, in agreement with the institutional regulations (Art. 8, University Medical Center Utrecht Biobank Regulations; version June 19, 2013). The synovial fluid used consisted of pooled residual synovial fluid from clinical joint aspirations in four patients with rheumatoid arthritis (*n* = 1), juvenile arthritis (*n* = 1), and unknown disease (*n* = 2). The synovial fluid residuals were visually inspected to confirm clear, non-purulent, non-haemorrhagic aspirates, kept frozen at − 80 °C, and were defrosted 24 h before being pooled to one volume of synovial fluid. The blood used was venous whole blood (haemoglobin 9.8 mmol/L), from a healthy male volunteer, collected into lithium-heparin tubes. The freshly withdrawn blood was mixed with the pooled synovial fluid residuals.

**Fig. 1:**
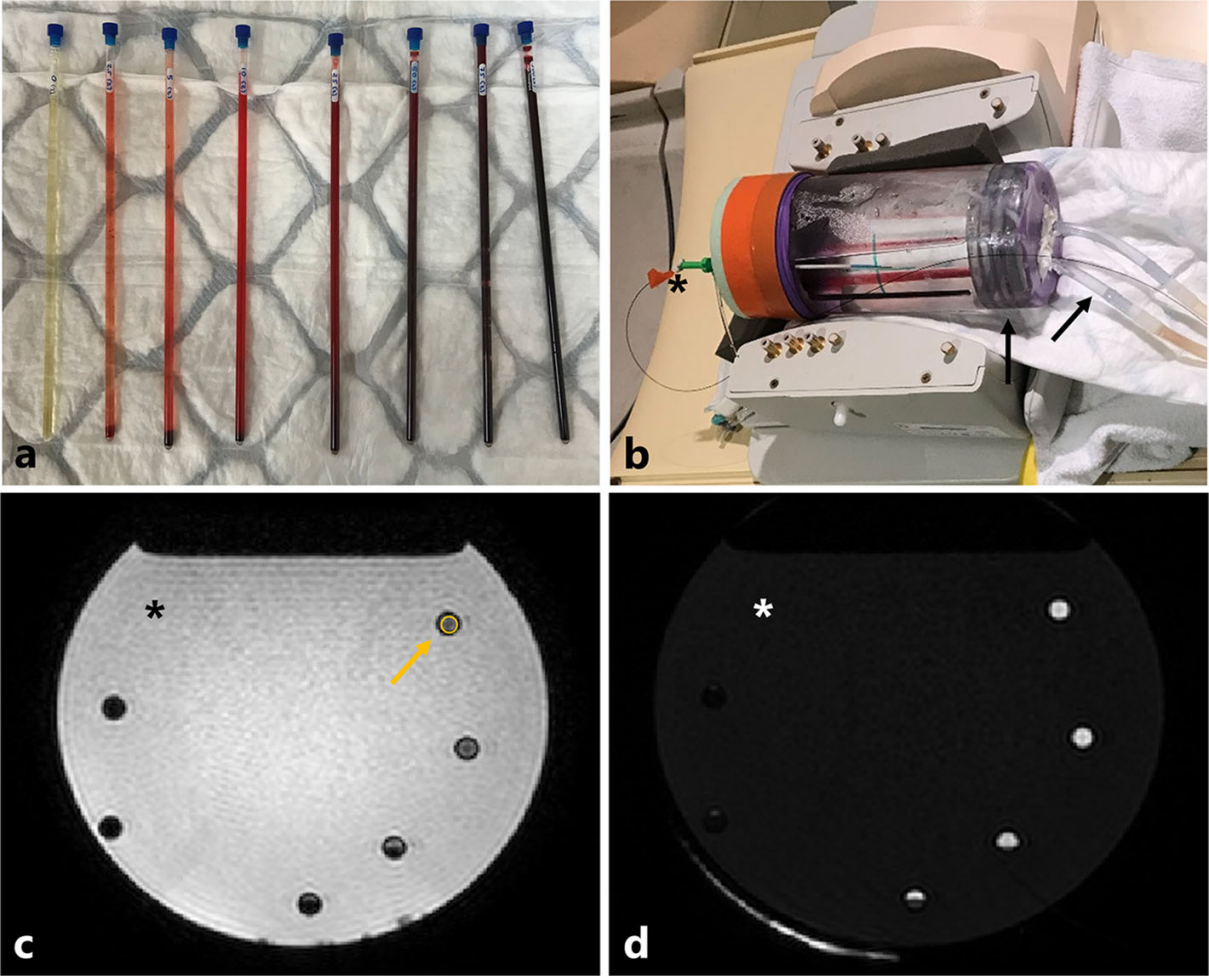
**a** Nuclear magnetic resonance tubes with different ratios of synovial fluid and blood. Blood percentage from left to right: 0%, 2.5%, 5%, 10%, 25%, 50%, 75%, and 100%. **b** Phantom setup: a cylindrical water-filled holder with six samples, horizontally placed in a 16-channel knee coil using a 3-T MRI system. The phantom was equipped with a fiberoptic thermometer (the asterisk in all images) and a heating system based on heat exchange between water in the heating system (black arrows) and the water or oil volume in the phantom. **c** Scan image from the T1 mapping sequence. For each tube, a region of interest was manually placed within the tube region (example in yellow). **d** Scan image from the T2 mapping sequence

Solutions with blood concentrations of 0, 2.5, 5, 10, 25, 50, 75, and 100% were prepared, and a volume of 3 mL was injected into nuclear magnetic resonance tubes (Wilmad LabGlass, WG-1000-8, Vineland, NJ, USA) (Fig. 1a). To ensure the blood was deoxygenated before starting the scanning sessions, the samples were kept at 37 °C for 24 h. Outside the scanner, the samples were kept in a stove at 37 °C and were manually shaken prior to scanning to maintain homogeneous mixtures.

### Phantom

The samples were horizontally placed in batches of six samples in a cylindrical holder filled with water at 1.5 T and 3 T or mark-oil at 7 T, to reduce magnetic susceptibility effects around the samples. Details can be found in the supplementary materials (Fig. S1). The temperature inside the phantom was monitored using a fiberoptic thermometer (Luxtron Corp., Santa Clara, CA, USA), and was kept stable around 37 °C during the scanning session using a heating system based on heat exchange between water in the heating system and the water or oil volume in the phantom (Fig. 1b).

### MRI data acquisition

The experimental data were acquired at 3 field strengths, using 1.5, 3, and 7 T systems (Philips Achieva, Best, The Netherlands). An 8-channel head coil and a 16-channel knee coil (Philips Achieva, Best, The Netherlands) were used at 1.5 and 3 T, respectively. A 32-channel head coil (Nova Medical, Wilmington, MA, USA) was used at 7 T. All acquisitions were performed in a time span of 25 h using the 1.5-T, the 3-T, and the 7-T scanners, in that chronological order.

### T1 mapping

Images for the T1 measurements were obtained using a multislice inversion recovery technique with a gradient-echo echo-planar imaging readout (Fig. 1c) [20]. The T1 mapping strategy consists of inverting the longitudinal magnetisation in the imaging volume using an adiabatic inversion pulse and subsequently sampling the recovery curve by changing the order in which the slices are acquired after the inversion. The details of the MRI protocols are reported in Table 1.

**Table 1:** MRI protocols for T1 mapping and T2 mapping with turbo spin-echo and sensitivity encoding acceleration, at 1.5, 3, and 7 T

Field strength	T1 mapping			T2 mapping*		
	1.5 T	3 T	7 T	1.5 T	3 T	7 T
Number of slices	11	11	11	5	5	5
Number of inversions	11	11	11	32	32	32
Voxel size (mm)	0.8	0.8	0.8	0.8	0.8	0.8
Field of view (mm)	128 × 128	128 × 128	128 × 128	128 × 128	128 × 128	128 × 128
Slice thickness (mm)	5	5	5	5	5	5
Inversion time spacing (ms)	315	315	907	–	–	–
Repetition time (ms)	10,000	10,000	10,000	3000	3000	7800
Echo time (ms)	7.8	7.8	4.33	20–950	20–950	30–960
Flip angle	15°	15°	15°	90°	90°	90°
EPI frequency encoding bandwidth (Hz)	614.6	614.6	1179	–	–	–
Readout bandwidth (Hz/pixel)	77.1	77.1	144.2	227	227	208
EPI factor	5	5	5	–	–	–
Parallel reduction factor in-plane	2	2	2	2	2	2
Scan duration (s)	220	220	220	252	168	632

*EPI* Echo-planar imaging, *with turbo-spin echo and sensitivity encoding acceleration

### T2 mapping

T2 mapping was performed using a multi-slice multiple spin-echo sequence, where images are acquired as the signal decays after the initial slice excitation (Fig. 1d). The different echo times (TEs) are separated by the echo spacing. To limit the scan duration, different acceleration techniques were applied: turbo-spin echo (TSE), echo-planar imaging, and a combination of TSE acceleration with sensitivity encoding (SENSE). The details of the sequences are reported in Table 1.

For T2 measurements, a parameter of particular significance in the pulse sequence is the echo spacing [21]. To verify that measured T2 values were independent of the echo spacing, four additional scans with different echo spacing (echo spacing 10, 20, 30, and 40 ms, respectively) were acquired at 1.5 T (information available in the Supplementary material).

### Image analysis

#### T1 and T2 fitting

Image processing was done offline using MatLab 2018a (MathWorks, Natick, MA, USA). T1 and T2 maps were obtained by voxelwise fitting the fit functions to the signal intensity (*S*) from the data points using a Levenberg-Marquardt non-linear least squares method. To obtain T1, the signal was fitted as *S* = *S*_0_ (1 − 2 e^−TI/T1^), where *S*_0_ is the spin density and TI is the inversion delay. To obtain T2, the signal was fitted as *S* = *S*_0_ (e^−TE/T2^), where TE is the echo time [22].

#### Postprocessing

The quantitative evaluation was based on the mean T1 or T2 values from regions of interest (ROIs) within each tube. The T1 and T2 standard deviation was assessed using the spatial standard deviation over the voxels inside the ROIs placed on the maps. Normally distributed data were reported as mean values with standard deviations. The normality of the data was evaluated by the Kolmogorov-Smirnov test. Finally, for each field strength, the mean T1 and T2 across the scan batches were calculated for different blood concentrations. To determine the lowest blood concentration that could be reliably measured by the T1 and T2 mapping, a method based on the guideline EPI17, by Clinical and Laboratory Standards Institute [23], was used. The synovial fluid, i.e. 0% blood concentration sample, was used as a blank sample. The limit of blank (LoB) was determined according to the guideline EPI17. The limit of detection (LoD) definition used by the Clinical and Laboratory Standards Institute was adapted to allow the discrimination of the highest apparent T1 or T2 likely to be reliably distinguished from the LoB among different blood concentrations. The LoD was determined by the following:

$$ \mathrm{LoD}={\mu}_{x\%\mathrm{blood}}+1.645\ {\sigma}_{x\%\mathrm{blood}} $$

where *μ*_*x* % *blood*_ is the mean T1 or T2 and *σ*_*x* % blood_ is the standard deviation of T1 or T2 for a given blood concentration of *x*%, fulfilling the condition of LoD < LoB. The blood concentration in percent corresponding to the LoD was defined as the detection threshold.

### Interrater reliability and agreement

To estimate the reproducibility of the measurements on the obtained T1 and T2 maps, all ROIs were independently determined by two raters, both relatively inexperienced in clinical radiology (FL, 4 months clinical radiology; BL, 4 years investigative radiology). Interrater reliability was assessed by intraclass correlation coefficient (ICC) for T1 and T2 mapping at the clinically available field strengths (1.5 and 3 T). ICC values range from 0 to 1, where values < 0.5, between 0.5 and 0.75, between 0.75 and 0.9, and > 0.90 indicate poor, moderate, good, and excellent reliability, respectively. ICC estimates and their 95% confidence intervals (CI) were calculated using the Statistical Package for the Social Sciences (IBM SPSS Statistics for Windows, version 26.0.0.1, Armonk, NY, USA) based on a single rater, absolute agreement, 2-way random effects model. Interrater agreement was assessed by the interrater limit of agreement (LoA) and graphically displayed in the Bland-Altman method with plots [24] for the previously mentioned sequences and field strengths.

## Results

Imaging was successful at all field strengths. Scan images for T1 mapping presented Gibbs ringing artefacts at 7 T, which hampered the analysis of the 75% and in part of the 100% blood concentration samples. Moreover, it was not possible to measure T2 values for blood concentration above 25% at 7 T because of the complete signal loss due to the short T2 of blood. A temperature between 35 and 38 °C was maintained in the phantom during the scanning sessions. Blood clot formation and sedimentation were observed to some extent in the samples during scanning.

### T1 measurements

**Fig. 2:**
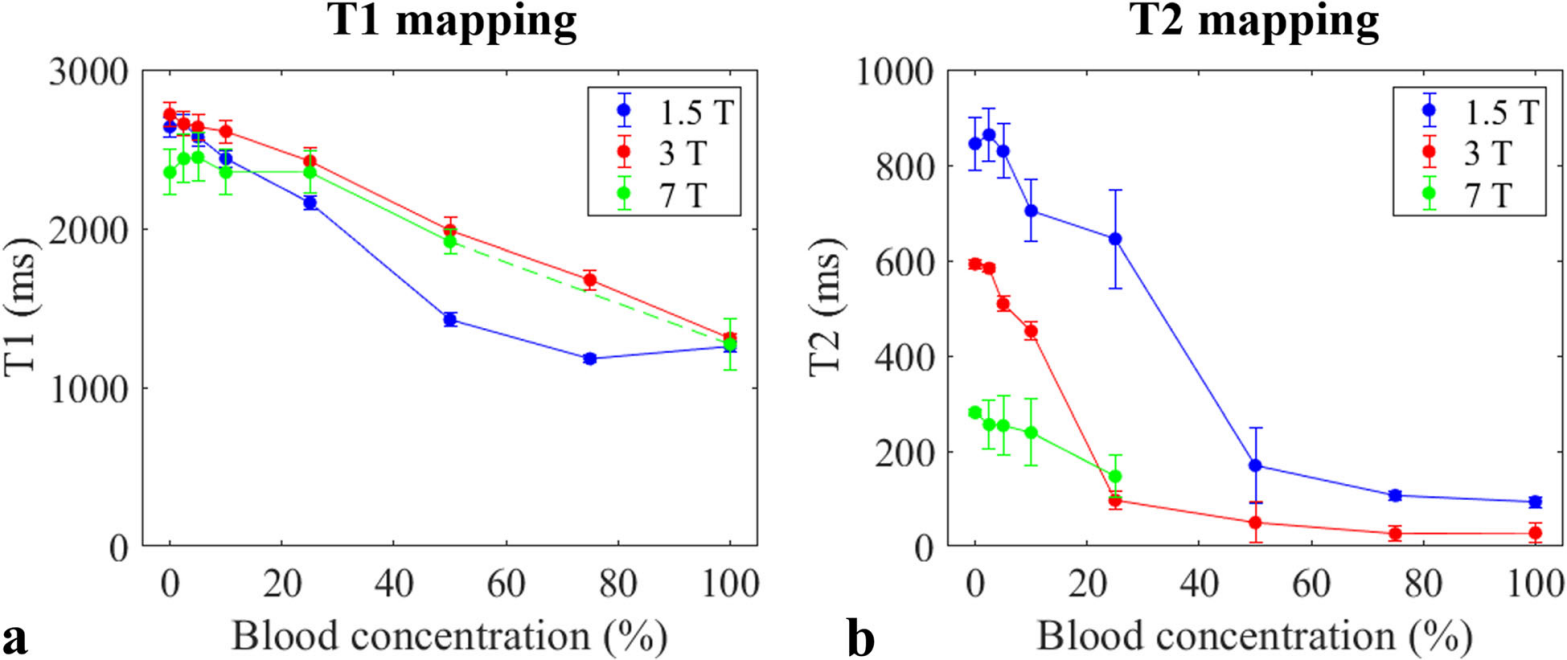
Mean T1 and T2 value estimates for the different blood concentrations, at 3 field strengths. For 7 T, some of the estimates were not available, because artefacts or relaxation times were too short to be measured

The mean T1 values as a function of blood concentration for each field strength are shown in Fig. 2. Overall, the estimated T1 values showed an inverse dependence on the blood concentration for all field strengths. The blood detection thresholds for T1 were ≥ 10% at 1.5 T, ≥ 25% at 3 T, and ≥ 50% at 7 T. The T1 estimates for blood and synovial fluid, the LoB (ms), the LoD (ms), and the blood detection threshold (blood percentage) are reported in Table 2.

**Table 2:** Experimentally estimated T1 and T2 values for synovial fluid, blood, LoB, LoD, and blood detection threshold at 1.5, 3, and 7 T

	Field strength	Relaxation time synovial fluid (ms)	Relaxation time blood (ms)	LoB (ms)	LoD (ms)	Blood detection threshold
T1	**1.5 T**	2641 ± 60	1258 ± 33	2541	2526	≥ 10%
	**3 T**	2719 ± 72	1310 ± 33	2600	2560	≥ 25%
	**7 T**	2355 ± 145	1272 ± 160	2117	2040	≥ 50%
T2	**1.5 T**	845 ± 56	93 ± 11	753	298	≥ 50%
	**3 T**	592 ± 13	28 ± 20	579	546	≥ 5%
	**7 T**	281 ± 5	Not measurable	272	218	= 25%

Results are reported as mean values, and the standard deviations as data were normally distributed (Kolmogorov-Smirnov test, *p* > 0.05)

*Blood* blood concentration 100%, *Blood detection threshold* blood concentration corresponding to the LoD, *LoB* limit of blank, *LoD* limit of detection, *synovial fluid* blood concentration 0%

### T2 measurements

**Fig. 3:**
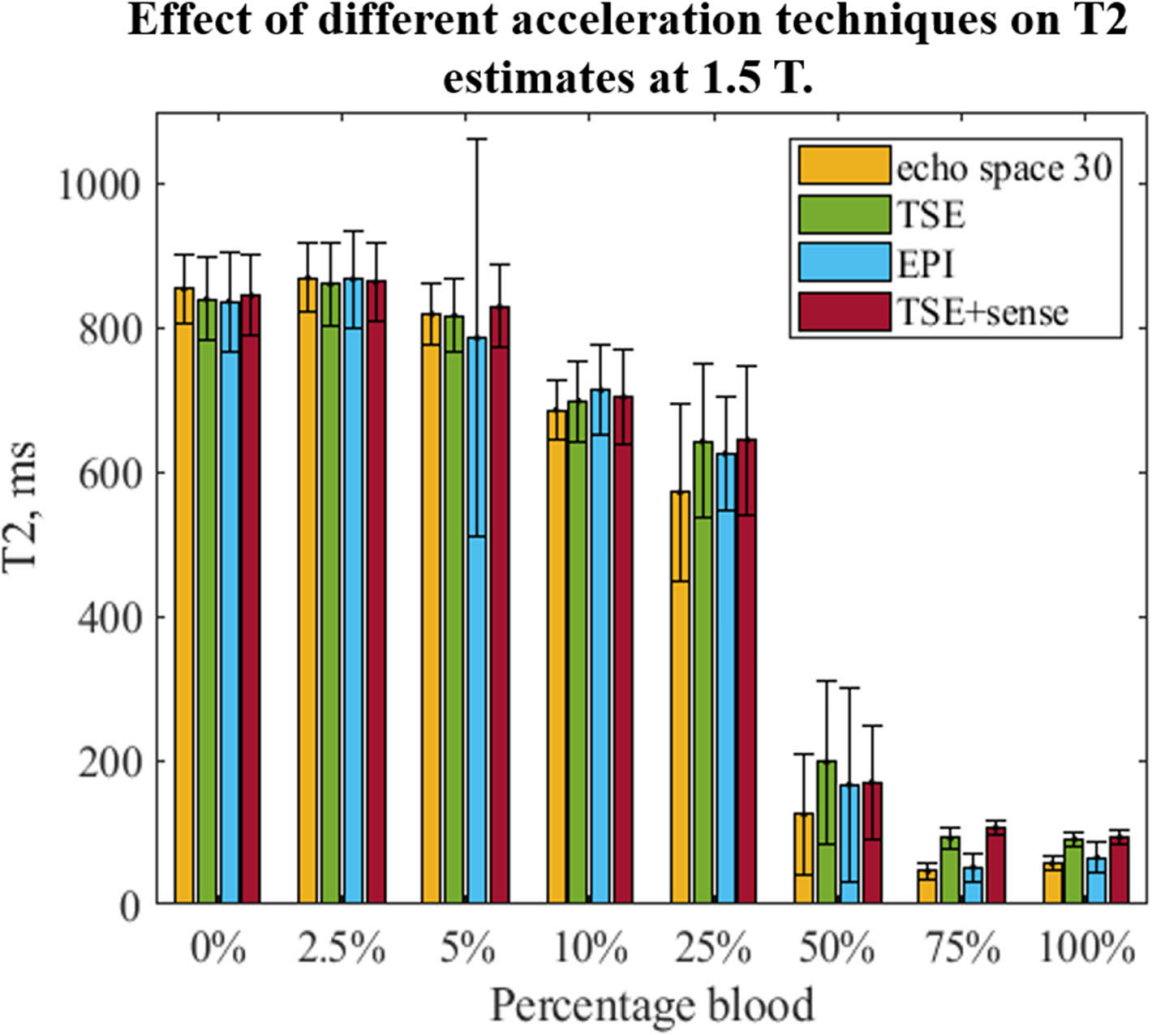
Effect of different acceleration techniques on T2 estimates at 1.5 T. T2 exhibited an inverse dependence on the blood concentration with all acceleration techniques. The three accelerated T2 mapping methods showed good agreement with the reference scan (echo spacing 30 ms), except from the 5% blood concentration measurement using the EPI acceleration technique. EPI, echo-planar imaging; TSE, turbo spin-echo; TSE+SENSE, turbo spin-echo with sensitivity encoding

At 1.5 T, different protocols for T2 mapping were employed (Fig. 3). With all the protocols, the T2 exhibited an inverse dependence on the blood concentration. The three accelerated T2 mapping methods showed good agreement with the reference scan (echo spacing 30 ms) in almost all cases. A large T2 standard deviation was identifiable for the 5% blood concentration with the EPI acceleration technique.

The mean T2 values for different blood concentrations using multi-slice TSE/SENSE-accelerated sequences at each field strength are shown in Fig. 2. The blood detection thresholds for T2 were ≥ 50% at 1.5 T, ≥ 5% at 3 T, and equal to 25% at 7 T. The T2 estimates for the blood and synovial fluid, the LoB (ms), the LoD (ms), and the blood detection threshold (blood percentage) are reported in Table 2.

### Interrater reliability and agreement

**Fig. 4:**
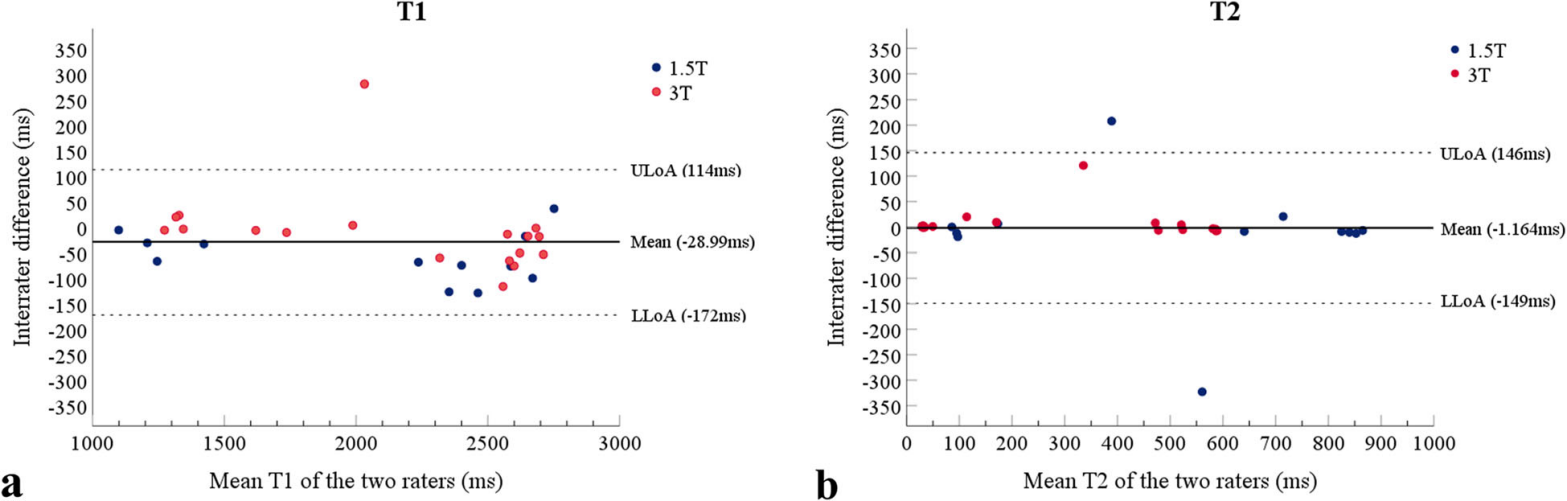
Bland-Altman plots for interobserver agreement regarding T1 and T2 measurements at 1.5 and 3 T. Horizontal dashed lines indicate the upper and lower limit of agreement from the mean by the two observers (ULoA/LLoA). For T1 measurements, the mean was − 28.99 ms, with a limit of agreement (LoA) equal to 143 ms. For T2 measurements, the mean was − 1.164 ms with LoA equal to 148 ms

Interrater reliability as assessed by the ICC was excellent for both T1 (0.991, 95% CI 0.980–0.996) and T2 (0.969, 95% CI 0.937–0.985) measurements at 1.5 and 3 T. The interrater LoA, which reflects the deviation of the measurements performed by the two observers compared to the mean value, was 143.15 ms for T1 and 147.51 ms for T2 (Fig. 4).

## Discussion

Our study presented T1 and T2 mapping MRI protocols aimed at detecting low blood concentrations in joints. We showed that different concentrations of deoxygenated blood and synovial fluid can be identified *in vitro* using quantification of the relaxation times at 1.5, 3, and 7 T. At all three field strengths, T1 and T2 relaxation times decreased as the blood concentration increased. Moreover, we were able to determine the detection thresholds of the T1 and T2 mapping approaches, i.e. the lowest blood concentration likely to be reliably distinguished from synovial fluid. For T1, the detection threshold was 10% at 1.5 T, 25% at 3 T, and 50% at 7 T. For T2, the detection threshold was 50% for 1.5 T, at 5% for 3 T, and is at least equal to 25% for 7 T.

We were unable to identify quantitative studies differentiating T1 and T2 for blood and synovial fluid at 1.5, 3, and 7 T. However, previous studies have reported on the relaxation times of blood and/or synovial fluid at various magnetic field strengths in *in vitro* and *in vivo* settings (Table 3). Our estimates of synovial fluid were in line with the relaxation times reported by previous works [25, 28]. Variations can be attributed to the intersubject variability of relaxation times in the synovial fluid [32, 33] or to the differences in the pulse sequence parameters, e.g. type of sequence, acquisition techniques, T1 and T2 estimation method, and echo times [25, 26, 28]. As for blood relaxation times, previous studies reported higher values than ours at 1.5, 3, and 7 T [26, 27, 30, 31]. This discrepancy probably results from a higher blood oxygen level [29, 31]: up to 95% oxygenated blood for reported values in the other studies [26], compared to fully deoxygenated blood in this study. At 7 T, estimates in the blood appeared to be different. However, the study at 7 T should only be considered as a proof of concept.

**Table 3:** T1 and T2 relaxation times of blood and synovial fluid at different field strengths: a comparison of the experimental values with literature values

	Field strength	Synovial fluid			Blood		
		Experimental values	Literature values	Author	Experimental values	Literature values	Author
**T1 (ms)**	**1.5 T**	2641 ± 60	2850 ± 280	Gold [25]	1258 ± 33	1440 ± 120	Stanisz [26]
						1480 ± 61	Zhang [27]
	**3 T**	2719 ± 72	3620 ± 320	Gold [25]	1310 ± 33	1932 ± 85	Stanisz [26]
			2564 ± 270	Jordan [28]		1649 ± 68	Zhang [27]
						1584 ± 5	Lu [29]
	**7 T**	2355 ± 145	4813 ± 700	Jordan [28]	1272 ± 160	2212 ± 53	Dobre [30]
						2087 ± 131	Zhang [27]
**T2 (ms)**	**1.5 T**	845 ± 56	1210 ± 140	Gold [25]	93 ± 11	290 ± 30	Stanisz [26]
	**3 T**	592 ± 13	767 ± 50	Gold [25]	28 ± 20	275 ± 50	Stanisz [26]
			652 ± 113	Jordan [28]		60 ± 6	Krishnamurty [31]
	**7 T**	281 ± 5	324 ± 60	Jordan [28]	Not measurable*	21 ± 4	Krishnamurty [31]

*Experimental values* Values measured in this study in region of interests within the synovial fluid (0% blood) and 100% blood tubes reported as means and standard deviations, *Literature values* Mean values found in other studies with corresponding standard deviations, *T2 times too short to be measured

Furthermore, we observed a lower blood detection threshold with T1 mapping at 1.5 T than at 3 T and the opposite for T2 mapping. This behaviour can be related to the compartmentalisation of paramagnetic haemoglobin in the blood cells, which is reminiscent of the way superparamagnetic iron oxide particles are distributed. The relaxation effects of these iron oxide contrast agents are strongly dependent on the physical characteristics of the individual nanoparticles and are influenced by their local concentration as well as the applied field strength [34]. In our case, a higher detection threshold is caused by a higher mean value and a bigger standard deviation. T1 measurements at 1.5 T were more precise than at 3 T, and the T1 relaxivity (the inverse of the relaxation time, i.e. 1/T1) of clustered paramagnetic compounds is higher at 1.5 T than at 3 T [35, 36]. Thus, due to a combination of stronger T1 effect and smaller T1 standard deviation at 1.5 T compared to 3 T, the LoD for T1 is lower at 1.5 T. The opposite can be observed for T2, where the effect of paramagnetic compartments, such as blood cells or iron oxide, increases with field strength [34, 36].

The current study reports on the performance of the developed MRI protocols in a phantom. Therefore, these results cannot be readily translated into patient settings. However, the observed trends and values give useful indications of T1 and T2 quantification in patients since the phantom mimics the conditions in a human joint. Firstly, the small volume of the scanned samples resembles the volume of synovial fluid in joints without large effusion. Secondly, the phantom was scanned at a constant temperature of 37 °C to simulate the intra-articular temperature. Thirdly, deoxygenated blood was used for the preparation of biological samples to ensure a stable oxygenation level throughout the scan session. Deoxygenated intra-articular blood is also expected in clinical and research settings after the expected patient delay between bleed onset and presentation at the clinic.

Nevertheless, the nature of the biological fluids employed in this study introduces some challenges on the experimental side. The blood used showed clot formation and sedimentation in the samples during scanning, despite heparinisation of the blood and the manual mixing before scanning. As a result of an inhomogeneous distribution of blood, observed T1 and T2 relaxation times might not fully correspond to values of homogeneous mixtures. However, clot formation and sedimentation can be expected to some extent *in vivo* as well. Therefore, imprecision of measured values *in vivo* can be expected too and cannot be prevented either. Furthermore, the reported T1 and T2 relaxation times were measured for blood with a haemoglobin level of 9.8 mmol/L, and relaxation times could vary with differences in haemoglobin levels.

The synovial fluid used came in part from patients with known inflammatory diseases. This might have increased the count of inflammatory cells in the synovial fluid mixture compared to the synovial fluid of patients without ongoing inflammatory joint processes. However, the concordance of our results to the values in the literature suggests that the effect of this increase in inflammatory cells on the relaxation time measurements can be considered minimal [25, 28].

From a technical perspective, the methods presented in this phantom study could be suitable for clinical settings, since a scan duration below 5 min of one T1 or T2 mapping sequence allows the use in clinical research and practice as part of the MRI protocol.

Additionally, the MRI sequences were shown to be sufficiently robust to be translated to different field strengths. The MRI protocols were initially developed at 1.5 T. Translation to 3 T was straightforward and effective. However, this choice led to compromises at 7 T. The echo times and spacing chosen for T2 measurements turned out to be suboptimal for estimating the short T2 at high blood concentrations at 7 T. Therefore, this study only shows a proof of concept of T2 measurements at 7 T.

At the clinically relevant magnetic field strengths, 1.5 and 3 T, the high ICC coefficient showed a high level of interrater reliability for the measurements. As supported by interrater agreement analysis, the T1 and T2 values estimated for each sample were independent from the rater as well.

What might be the potential use of these techniques in future patient studies or clinical practice? After *in vivo* validation, T1 and T2 mapping might be used for non-invasive differentiation between minor joint bleeding and no joint bleeding. The developed MRI sequences might be used to accurately detect low concentrations of blood in case of joint bleeding, whereas simple effusions suggest a non-bleeding aetiology. Therefore, the results of this study might eventually be used for research purposes or even in clinical cases as an objective reference standard to verify or rule out recent joint bleeding.

However, factors of variability should be investigated further, and some precautions need to be adopted for establishing the optimal usage of these sequences in clinics. Since the relaxation times of synovial fluid and blood depend on different conditions [25, 26, 28, 37–39], the effect of changes in variables as field strength and MRI pulse sequences, inter-patient differences in synovial fluid consistency and haemoglobin level, and time between bleed onset and scanning—and thereby differences in the blood oxygenation level—should be taken into account when interpreting the results.

To summarise, this study is providing different tools for the differentiation between simple and haemorrhagic joint effusion *in vitro*. *In vivo* validation of the MRI protocols is needed to establish the use in patients. A study in persons without joint bleeding may determine reference values for T1 and T2 of the synovial fluid *in vivo* that will enable interpretation of T1 and T2 measurements in patients with suspected joint bleeding. Evaluation of a joint with (suspected) bleeding and the contralateral joint without bleeding as a reference may be useful for comparison. Furthermore, in daily practice, the MRI protocol should be adapted according to the MRI field strength and sequence availability. If a 3-T scanner is available, our results indicate that quantitative T2 mapping allows detection of the lowest blood concentration (equal to 5%). In case a 3-T scanner is not available, T1 mapping at 1.5 T allows for the detection of blood concentrations as low as 10%.

This study shows that MRI T1 and T2 mapping techniques may differentiate between simple and haemorrhagic effusion *in vitro.* The relaxometry protocols were able to detect low blood concentrations in the synovial fluid at clinically available field strengths (1.5 and 3 T). Especially T2 measurements at 3 T MRI appeared to be highly sensitive with the lowest detectable blood concentration of 5%. With limited acquisition times below 5 min, these T1 and T2 mapping techniques might be promising for accurate non-invasive discrimination between simple and haemorrhagic joint effusion *in vivo*, for example, in patients with bleeding disorders.

## Supplementary Information

**Additional file 1: Figure S1.** Detailed description of the position of the samples in the phatom in the different batches scanned. Percentages correspond to the blood percentage in the sample. a) First batch scanned at all field strenghts. b) Second batch scanned at all field strengths. c) Third batch scanned at 3T. **Figure S2.** (A) Signal intensity in the ROI vs IR delays for the 0% blood tube at 3T: the experimental data are indicated with blue ● and the fitting with a red line. (B) Analysis of the residuals: each ● represents the residual of each experimental data point from the one predicted with the fitting. The mean of the residual is also reported as dotted line. **Figure S3.** T2 mapping at 1.5T with different echo spacing on dependence on the blood concentration. **Figure S4.** T2 mapping at 1.5T (A) and 3 T (B) with a multi-slice accelerated scans and single slice non accelerated scans.

## Abbreviations

CI: Confidence intervals
EPI: Echo planar imaging
ICC: Intraclass correlation coefficient
LoA: Limit of agreement
LoB: Limit of blank
LoD: Limits of detection
MRI: Magnetic resonance imaging
ROI: Region of interest
SENSE: Sensitivity encoding
TE: Echo time
TSE: Turbo spin-echo
