# Supplementary material for: Detecting low blood concentrations in joints using T1 and T2 mapping at 1.5, 3, and 7 T: an in vitro study
Source: Eur Radiol Exp. 2021 Dec 2;5:51. doi: 10.1186/s41747-021-00251-z (PMC8636530; doi:10.1186/s41747-021-00251-z)
Supplement: Supplementary file 1 — Additional file 1: Figure S1. Detailed description of the position of the samples in the phatom in the different batches scanned. Percentages correspond to the blood percentage in the sample. a) First batch scanned at all field strenghts. b) Second batch scanned at all field strengths. c) Third batch scanned at 3T. Figure S2. (A) Signal intensity in the ROI vs IR delays for the 0% blood tube at 3T: the experimental data are indicated with blue ● and the fitting with a red line. (B) Analysis of the residuals: each ● represents the residual of each experimental data point from the one predicted with the fitting. The mean of the residual is also reported as dotted line. Figure S3. T2 mapping at 1.5T with different echo spacing on dependence on the blood concentration. Figure S4. T2 mapping at 1.5T (A) and 3 T (B) with a multi-slice accelerated scans and single slice non accelerated scans. [file 41747_2021_251_MOESM1_ESM.docx]

**ELECTRONIC SUPPLEMENTARY MATERIAL**

Samples were scanned in batches of 6 samples. Detailed information on the positioning of the samples in the phantom in the different batches is provided in Figure S1.

**
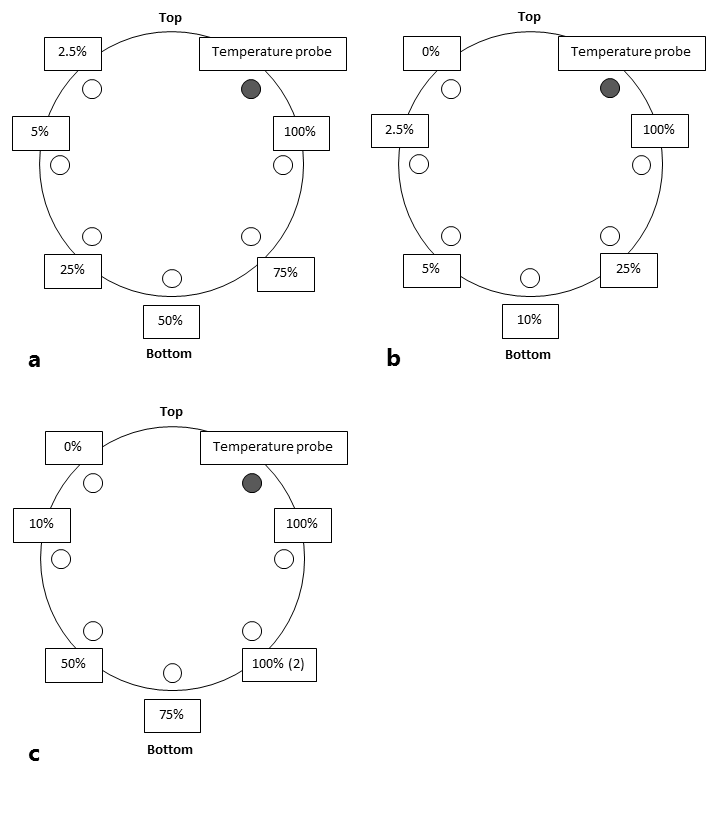
**

Figure S2 shows the plot of the signal intensities for the 0% blood tube, from the second batch scanned at 3T versus the IR delays: the data were fitted for estimating T1. A plot of the residuals is also reported: all the values rely in the interval between -0.2 and 0.15.

**Figure S1.** Detailed description of the position of the samples in the phatom in the different batches scanned. Percentages correspond to the blood percentage in the sample. **a)** First batch scanned at all field strenghts. **b)** Second batch scanned at all field strengths. **c)** Third batch scanned at 3T.


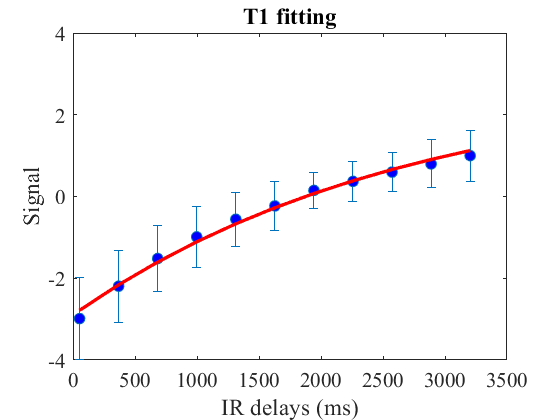

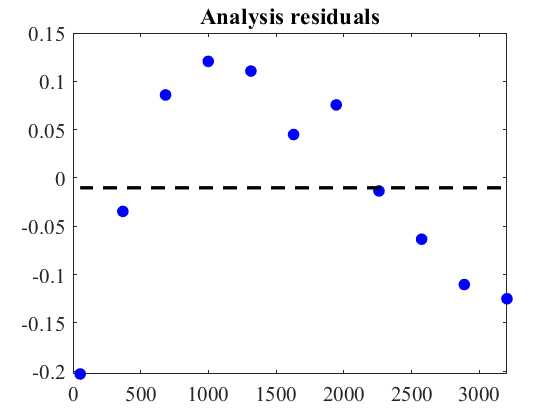


**(B)**

**(A)**

**Figure S2.** (A) Signal intensity in the ROI vs IR delays for the 0% blood tube at 3T: the experimental data are indicated with blue ● and the fitting with a red line. (B) Analysis of the residuals: each ● represents the residual of each experimental data point from the one predicted with the fitting. The mean of the residual is also reported as dotted line.


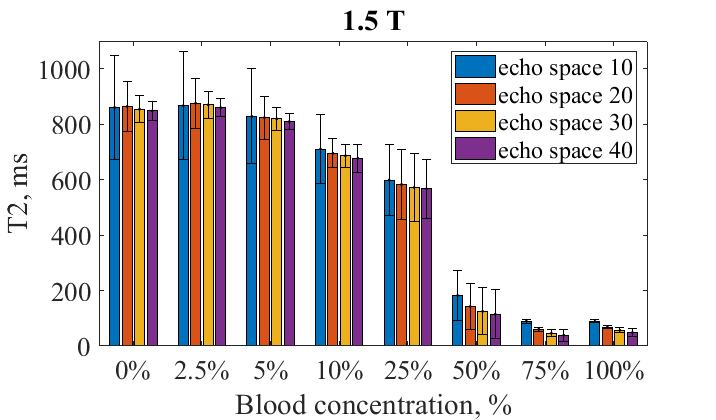
At 1.5 T, different MRI parameter setup were adopted for T2 mapping, as shown in Figure S3. With all the setups, the T2 exhibited an inverse dependence on the blood concentration. The performances of the T2 mapping with different echo spacings showed in almost all cases good agreement with the reference scan (echo space 30ms). Although the T2s estimated with echo space 10 showed most deviation compared to those obtained with echo space. 30, the dependence of T2 on blood concentration was similar for all echo spaces, suggesting that the actual echo space is not of primary interest, but should be the same between subjects (controls vs. patients).

**Figure S3.** T2 mapping at 1.5T with different echo spacing on dependence on the blood concentration.


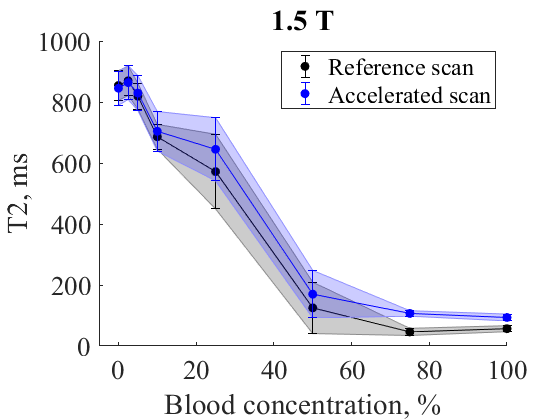

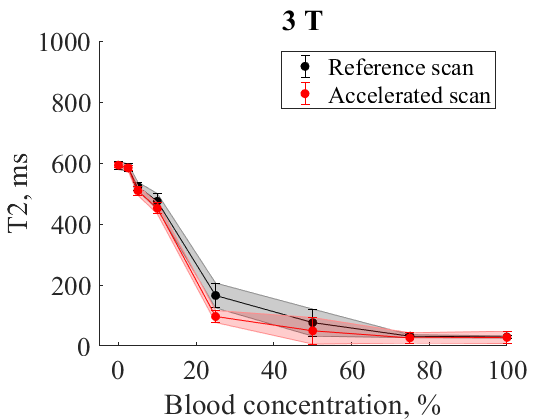
The accelerated TSE+SENSE multi-slice T2 mapping at 1.5T and 3T were compared with

**Figure S4.** T2 mapping at 1.5T (A) and 3 T (B) with a multi-slice accelerated scans and single slice non accelerated scans.

the single slice reference mapping without acceleration. The performances of the T2 mapping for both field strength showed comparable results with and without acceleration.

The blood detection thresholds for T2 were:

- ≥50% with accelerated scans and ≥10% with reference scans at 1.5T;
- ≥5% with both accelerated scans and reference scans at 3T.
